# Floral development of *Dieffenbachia* and the occurrence of atypical flowers in Araceae

**DOI:** 10.1186/1999-3110-55-30

**Published:** 2014-03-14

**Authors:** Denis Barabé, Christian Lacroix

**Affiliations:** 1 Institut de Recherche en Biologie Végétale, Université de Montréal, Jardin Botanique de Montréal, 4101 rue Sherbrooke Est, Montréal, H1X 2B2 Québec Canada; 2 Department of Biology, University of Prince Edward Island, 550 University Avenue, Charlottetown, C1A 4P3 PE Canada

## Abstract

**Background:**

The floral development of *Dieffenbachia seguine* (N. J. Jacquin) Schott is analysed in relation to the molecular phylogeny of the Spathicarpae by Cusimano et al. (Am J Bot 98:654–668, 2011).

**Results:**

The initiation of discoid floral primordia occurs acropetally on the surface of the spadix. Female flowers, atypical bisexual flowers, sterile male flowers, and male flowers share the same phyllotactic spirals on the spadix. Four or five stamen primordia are initiated simultaneously on the periphery of the male floral meristem. During early stages of stamen initiation, individual stamen primordia are connate at their base. In a synandrium, stamen fusion occurs very early during their developmental cycle with the stamens being already united laterally when longitudinal elongation occurs. The staminodes are also initiated on the periphery of the discoid floral primordium, and their number varies from four to six. The development of the fused staminodes will eventually form a longitudinal cavity in the center of the mature synandrode. The atypical flowers located in the intermediate zone range in morphology from aborted female flowers to rudimentary sterile male flowers with incomplete staminodes.

**Conclusions:**

The development of the female flower of *Dieffenbachia* Schott presents some similarities with atypical bisexual flowers of *Cercestis* Schott representing one of three types of aberrant flower forms in the family. From a developmental point of view and in the context of a phylogeny of the group, we believe that the presence of staminodes in the female zone constitutes a plesiomorphy in the tribe Spathicarpeae.

**Electronic supplementary material:**

The online version of this article (doi:10.1186/1999-3110-55-30) contains supplementary material, which is available to authorized users.

## Background

The flowers of the Araceae represent a good model for conducting comparative developmental studies at different hierarchical levels. At the structural level, the elongate spadix consisting of unisexual flowers in many genera offers the possibility to study the transition from female to male flowers from a developmental point of view. At the phylogenetic level, recent well resolved molecular phylogenies (Cabrera et al., 2008; Cusimano et al., 2011), allow us to integrate floral developmental morphology in the context of a rigorous systematic framework.

As the largest subfamily of the Araceae, Aroideae consists of 81 genera (Boyce and Croat, 2011). This group is generally characterised by unisexual flowers arranged in well-delineated zones on the spadix. The female flowers are located in the lower portion of the spadix and the male flowers (sterile and fertile) are found directly above them. Rare exceptions to this arrangement include *Calla* L., *Furtadoa* M. Hotta, and *Spathicarpa* Hook. To date species representing different genera with unisexual flowers have been investigated by a variety of authors from a perspective of floral anatomy or developmental morphology (Barabé et al., 2004a, 2008, 2012; Buzgó 1994; Barahona Carvajal, 1977; Carvell, 1989; Engler and Krause, 1912; Eyde et al., 1967; French, 1985; Fukai, 2004; Hotta, 1971; Mayo, 1986, 1989; Uhlarz 1982, 1986).

In many genera of Aroideae atypical flowers with both male and female characteristics are often found in the intermediate portion of the spadix between the female zone and the male zone (*Culcasia* P. Beauv., *Cercestis, Schismatoglottis* Zoll. & Moritzi Schott) or between the female zone and the sterile male zone (*Philodendron* Schott*,*) (Barabé, 2013). These atypical flowers have been referred to as « monströse Blüten » by Engler and Krause (1912). To date, three types of atypical bisexual flowers (ABFs) have been described: the *Philodendron* type, the *Cercestis* type (Barabé and Lacroix, 1999), and the *Schismatoglottis* type, *sensu* Barabé et al. (2004b).

In the *Philodendron* type, ABFs consist of functional carpels and staminodes inserted on a single whorl. In the *Cercestis* type, ABFs are characterized by a functional or non-functional gynoecium surrounded by 1 to 5 vestigial stamens on a separate whorl (Barabé and Bertrand, 1996, Figure Thirty). In the *Schismatoglottis* type, the intermediate zone located between the female and male zones consists of atypical flowers displaying a wide variety of forms ranging from more or less typical female flowers to aberrant, non-functional male flowers (Barabé et al., 2004b).

In a previous study, the distribution of atypical flowers of the *Cercestis* type and the *Philodendron* type in selected genera was correlated to a partial phylogeny of the Araceae (Barabé et al., 2002). It was shown that these two different developmental patterns of atypical bisexual flowers appear to have arisen several times during the evolution of the family. In Barabé et al. (2002), it was hypothesized, based on the presence of staminodes around the ovary, that the atypical flowers in the intermediate zone of the spadix of *Dieffenbachia* belonged to the *Cercestis* type. To confirm this, we performed a detailed comparison of the floral development of *Dieffenbachia* to that of *Cercestis* to determine which type of atypical flowers the sterile flowers in the intermediate zone of the spadix belong to. This point is particularly interesting because Gonçalves et al. (2007) used *Cercestis* as an outgroup in their molecular analysis.

The genus *Dieffenbachia* belongs to the monophyletic tribe Spathicarpeae, sensu Cusimano et al. (2011), Gonçalves (2005) and Gonçalves et al. (2007). Gonçalves et al. (2007) performed a detailed systematic analysis of the tribe Spathicarpeae which consists of 12 genera including *Dieffenbachia* (Table 1). All genera of the tribe Spathicarpeae are characterized by the presence of synandria, and a chromosome number of 17, two characters that constitute a synapomorphy of the tribe (Cusimano et al., 2011; Gonçalves et al., 2007). However, synandria are also present in other genera outside the Spathicarpeae, for example *Caladium*, *Syngonium,* and *Xanthosoma* (Cusimano et al., 2011). Until now, in the tribe Spathicarpeae, the development and anatomy of the inflorescence was studied only in the genus *Spathicarpa*(Uhlarz, 1982; Barabé et al., 1985). However, the early stages of development of the synandria were not described in detail in the work of Uhlarz (1982) who dealt mainly with the analysis of the fusion of spathe and spadix. Consequently, the ontogeny of synandria and synandrodes in this tribe remains unknown.

**Table 1:** **Characters of the inflorescence and flower of genera belonging to the tribe** ***Spathicarpeae*** **sensu Cusimano et al. (** 2011 **)**

Genus	Spadice fused to the spathe	Spadice female–male zones	Staminodes around female flowers	Synandrium	Sterile male zone
*Asterostigma*	+ or No	+	+	+	No
*Bognera*	+	+	No	+	± Free
Croatiella*	+	+	+	± Synandrium	No
*Dieffenbachia*	+	+	+	+	± Synandrode
*Gearum*	No	+	+	+	Synandrode
*Gorgonidium*	No	+	+	Free or ± synandrium	No
Incarum*	+	+	+	+	No
*Mangonia*	No	+	+	+	Synandrode
*Spathantheum*	+ (Entirely)	+	+	+	Male and female flowers mixed
*Synandrospadix*	+ or No	+	+	+	+ or bisexual flowers
*Spathicarpa*	+ (Entirely)	No	+	+	No
*Taccarum*	+ or No	+	+	+	No

The data are from Mayo et al. (1997) and (*) Gonçalves (2005).

+ means present; +/- means present or absent.

The initiation and development of synandria and synadrodes in the Araceae was documented in detail for the first time in the genus *Syngonium,* tribe Caladieae (Barabé et al., 2012). It was shown that during the early stages of development, the stamen and staminode primordia are clearly recognizable. The union of these appendicular organs occurs very early during the developmental cycle of a synadrium or synandrode. The further growth of the fused stamens or staminodes eventually results in the formation of a synandrium or a synandrode.

Although the mature forms of synandria look similar among genera, it does not necessarily imply that the mode of development is the same in distantly related taxa. In that context, the analysis of the floral development of *Dieffenbachia* will allow us to compare the mode of development of synandria in two different tribes: Spathicarpeae and Caladieae.

The genus *Dieffenbachia* is an interesting taxon, as far as the diversity of atypical bisexual flowers is concerned, that can be used to infer the changes that may have taken place in developmental morphology during evolution. The analysis of floral development in *Dieffenbachia* allows us to document the floral development of Spathicarpeae in the context of a well resolved molecular phylogeny (Cusimano et al., 2011). In the present study, we assess whether the floral development of *Dieffenbachia* can be integrated into the developmental patterns already characterized in the subfamily Aroideae.

The specific goals of this study are: (1) to document the mode of development of synandria and synandrodes in *Dieffenbachia*; (2) to compare the development of female and sterile flowers of *Dieffenbachia* with other genera that have atypical bisexual flowers; and (3) to further characterize the range of floral developmental morphologies in the subfamily Aroideae.

## Methods

Specimens of *Dieffenbachia seguine* (N. J. Jacquin) Schott were collected in French Guiana (Kourou, Grounds of ECOFOG) in May 2011. A total of 28 inflorescences at various stages of development were fixed in FAA, formalin-acetic acid-alcohol (1:1:9 by volume), for a minimum of 24 hours and then stored in 70% ethanol. Inflorescences were dissected using a stereo microscope to expose the surface of the spadix. The spadix was dehydrated in a graded ethanol series to absolute ethanol. Dissected inflorescences were dried in a LADD model 28000 critical point dryer using CO_2_ as a transitional fluid, mounted on metal stubs, and grounded with conductive silver paint. Specimens were sputter coated 30 nm with gold/palladium using a Denton Vacuum Desk II sputter coater and viewed with a Hitachi TM3000 scanning electron microscope (SEM) with digital imaging capabilities at the University of Prince Edward Island.

## Results

### Morphology of mature flowers and inflorescences

**Figure 1:**
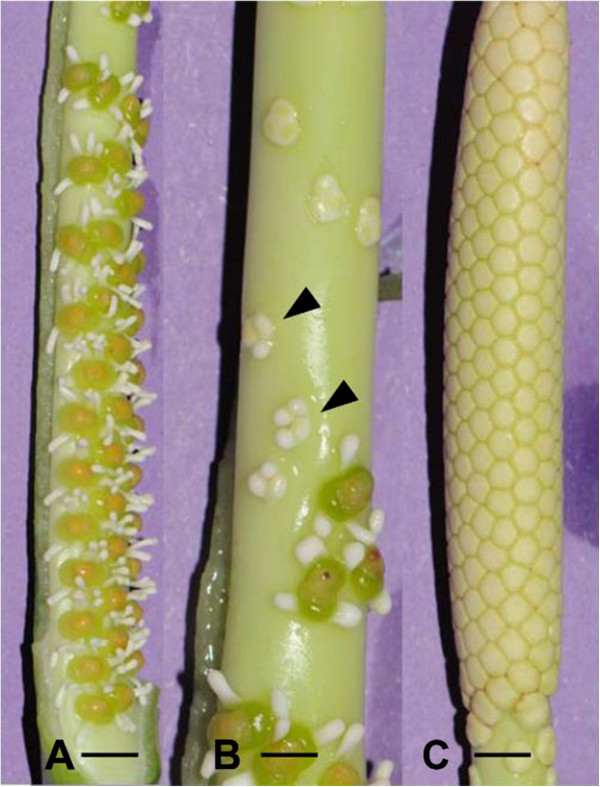
**Mature sections of the inflorescence. A**. Female zone at base of inflorescence. Scale bar = 0.3 cm. **B**. Intermediate zone with atypical flowers (arrowheads) situated between female and male zones. Scale bar = 0.2 cm. **C**. Male zone in the upper portion of the inflorescence. Scale bar = 0.5 cm.

*Dieffenbachia seguine* is a more or less rhizomatous plant that usually grows in open undergrowth. The inflorescences, generally 2–4 per sympodium, are situated in the upper portion of the plant. The green spathe (13–22 cm long) is distinctly constricted in the median portion, between the lower part and limb. The spadix axis (10–20 cm long) is green and has approximately the same length as the spathe. The female flowers occupy the lower portion (4–6 cm) of the spadix (Figure 1A), and the male flowers the upper portion (6 – 9 cm) (Figure 1C). The female zone is completely adnate to the spathe on one side. Between those two zones there is an intermediate zone (1 – 3 cm) consisting of a few loosely packed sterile flowers (Figure 1B). The male zone represents approximately 60% of the total length of the spadix, and the female zone 35%.

**Figure 2:**
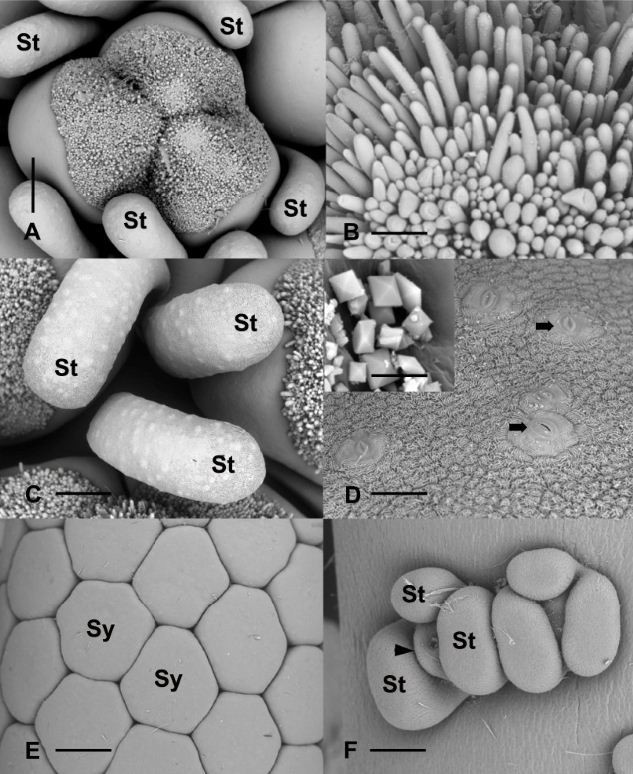
**Detail of mature flowers. A**. Female flower consisting of a 3 locular ovary (center) surrounded by staminodes (St). scale bar = 375 μm. **B**. Stigmatic surface showing papillae. Scale bar = 61 μm. **C**. Top view of staminodes (St). Light colored protuberances correspond to stomata. Scale bar = 300 μm. **D**. Higher magnification of stomata on the epidermal surface of staminodes (arrows). Scale bar = 38 μm. Insert, close-up of calcium oxalate crystals on the epidermal surface. Scale bar =3 μm. **E**. top view of synandria (Sy). Scale bar = 375 μm. **F**. Two atypical flowers in the intermediate zone. St, staminode; arrowhead, incompletely developed gynoecium. Scale bar = 101 μm.

Mature female flowers consist of a subglobose to ovoid sessile ovary ([1] 2 [3] -locular) surrounded by a whorl of 3 – 4 clavate staminodes (Figure 2A,C). The stigmas are divided into two or three parts coinciding with the number of locules in the ovary (Figure 2A), and bear elongate papillae (Figure 2B).

Male flowers consist of 4–5 connate stamens forming a rhomboid to hexagonal synandrium that is truncated apically (Figure 2E). The anthers have a thick connective and open by pores located apically on the thecae. The stamens form a regular pattern on the surface of the spadix. In *D. seguine,* the surface of the epidermis of the stamens and staminodes is covered with stomata (Figure 2D).

In a transition zone between female flowers and male flowers, reproductive organs ranging from sterile female flowers, near the female zone, to aberrant synandrodes at the base of the male zone are present (Figures 1B, and 2F).

The presence of calcium oxalate crystals is observed on the surface of synandria, synandrodes and stigmata (Insert, Figure 2D).

### Development

#### Development of the inflorescence

**Figure 3:**
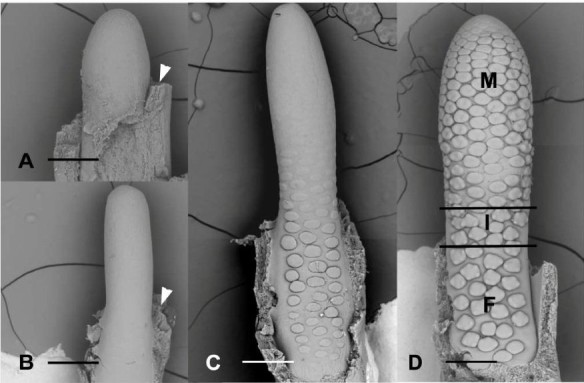
**Early stages of development of flowers and inflorescences. A**, **B**. Early stages of initiation of the inflorescence. Arrowhead, adnation of spathe (removed) at the base of the inflorescence. Scale bars = 188 μm **(A)**; 375 μm **(B)**. **C**. Acropetal initiation of more or less circular floral primordia along the spadix. Scale bar = 375 μm. **D**. Stage at which unisexual floral primordia begin to form their floral organs. F, female zone. I, intermediate zone. M, male zone. Scale bar = 375 μm.

During early stages of development, the spadix primordium has an obovoid form. The diameter of the spadix is larger in the median part of the structure (Figure 3A). At this stage, the spadix is already adnate to the spathe along its lower half, which will correspond later to the female zone (Figure 3A,B). During later stages of development, the spadix will develop an obcylindrical shape (Figure 3B). The initiation of more or less discoid floral primordia occurs acropetally when the spadix is approximately 2 mm long (Figure 3C), which represents 2% of its mature length. After the initiation of floral organs, the different zones of the spadix are recognizable: the female zone at the base, the male zone in the upper part, and the intermediate zone occupying the median portion (Figure 3D). At the median portion of the inflorescence corresponding to the zone where atypical flowers are formed, the diameter of the inflorescence is narrowest (Figure 3D). There is no terminal flower at the tip of the spadix. During early stages of development, there is no discontinuity between the different zones of the spadix (Figure 3D). However, the packing of floral primordia is not uniform along the spadix: male primordia (top) are more densely packed than female primordia (bottom) (Figure 3D). Although the floral primordia are more or less densely packed on the surface of the spadix, it is not possible to recognize distinct continuous parastichies (Figure 3D).

#### Female flowers

**Figure 4:**
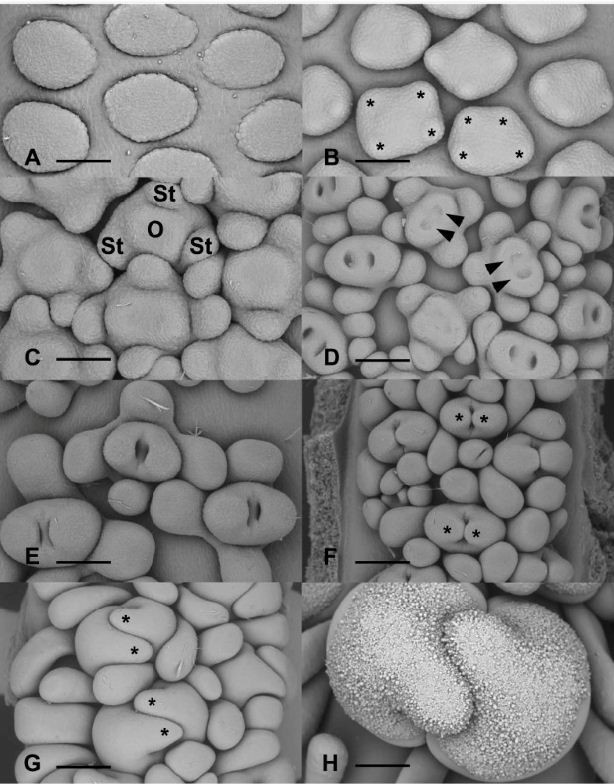
**Developmental stages of female flowers. A**. Floral primordia prior to the initiation of staminodes. Scale bar = 75 μm. **B**. Initiation of staminodes (asterisks). Scale bar = 83 μm. **C**. Initiation of the gynoecium (O) at the center of the floral primordium. St, staminodes. Scale bar = 83 μm. **D**. Formation of the locules (arrowheads). Scale bar = 150 μm. **E**. Older stage of development showing the closure of locules. Scale bars = 125 μm. **F**. Early stage of formation of stigma (asterisks). Scale bars = 188 μm. **G**. Elongation and imbrications of stigma (asterisks). Scale bars = 300 μm. **H**. Early stage of initiation of papillae on stigma. Scale bar = 375 μm.

During early stages of development, female floral primordia are oval with the longer axis of the flower oriented transversely to the main axis of the spadix (Figures 3D and 4A,B). Three to four (rarely five) staminodial primordia are the first appendages to be initiated simultaneously at the periphery of the floral primordium (Figure 4B), followed rapidly by the initiation of the ovary (Figure 4C). After their initiation, the staminodes will rapidly acquire their typical clavate form (Figure 4D-G). The development of the two (rarely three) locules begins with the appearance of one, two or three shallow cavities on the gynoecial primordium (Figure 4C,D). The growth of the ovary wall, formed by the fusion of adjacent carpels, defines the locules (Figure 4D,E). The apical portion of the carpels grows to form individual stigmas (Figure 4F,G). Before the appearance of papillae, the tips of each carpel become imbricated (Figure 4G). The initiation of papillae begins in the median portion of the carpel and eventually covers the entire stigmatic surface (Figure 4H). During early stages of development, the stigmatic papillae are subglobose but at maturity they are generally elongate (Figure 2B). With the further growth of the gynoecium and staminodes, the floral primordia touch each other, and eventually occupy all the available space between flowers (Figure 4C,G).

#### Male flowers

**Figure 5:**
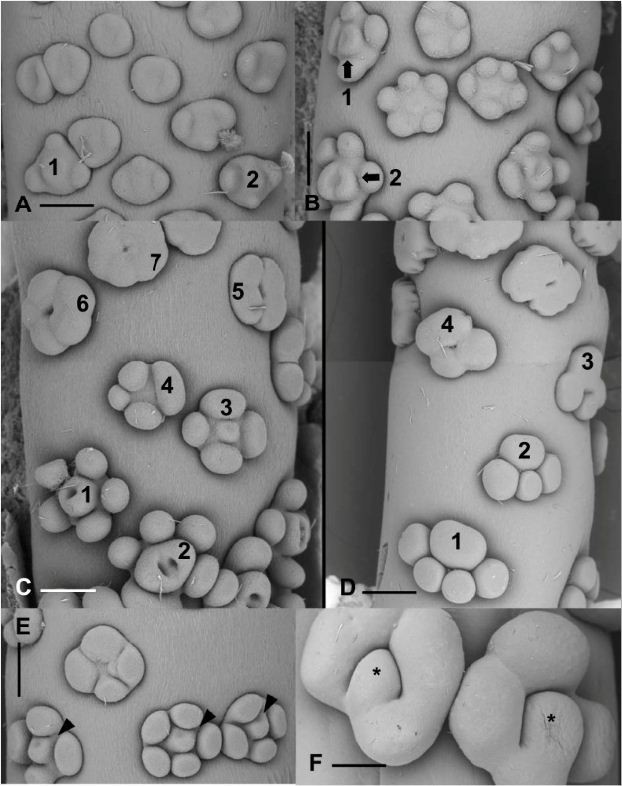
**Developmental stages of male flowers. A**. Initiation of stamens (asterisks) at the periphery of the floral primordium. Scale bars = 75 μm. **B**. Later stage of development of stamens (asterisks). Scale bars = 150 μm. **C**. Early stage of formation of synandria by the concrescence of stamen primordia (asterisks). Scale bar = 125 μm. **D**. Stage at which synandrium becomes a unit. Arrows, depression at the center of the synandrium. Scale bar = 150 μm. **E**. Nearly mature synandria with depression (arrow) still visible. Scale bar = 250 μm. **F**. Higher magnification of mature synandria. The central depression (arrow) remains visible on some flowers. Scale bar = 150 μm.

Male floral primordia are more or less circular or oval in form at initiation (Figure 5A). Four or five stamen primordia are initiated simultaneously on the periphery of the floral meristem (Figure 5A-C). The male flowers are compactly arranged throughout their development, and touch each other during later stages of development. The size of the stamens increases and they eventually occupy all the available space between flowers (Figure 5D,E). During early stages of stamen initiation, individual stamen primordia are connate at their base (Figure 5C). In a synandrium, the fusion of stamens occurs very early during their developmental cycle with the stamens being already united laterally when longitudinal elongation occurs. The further growth of the stamens leads to the formation of a more or less pentagonal or hexagonal-shaped synandrode with a depression in its center (Figure 5D-F). This cavity will disappear in nearly all mature flowers (Figures 1C and 5F).

#### Atypical flowers

**Figure 6:**
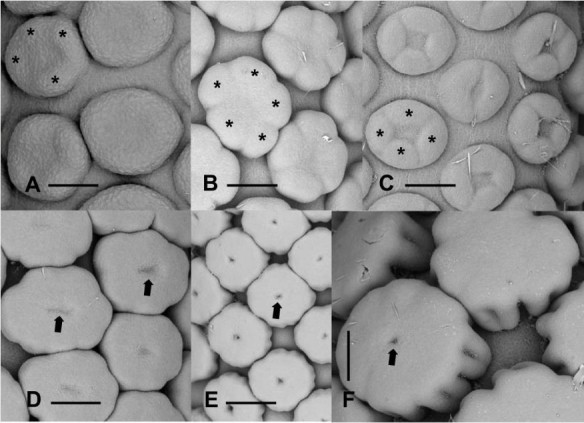
**Development of synandrodes and atypical flowers. A**. Atypical female floral primordia (1, 2). Scale bar = 150 μm. **B**. Atypical female flowers showing bicarpellate (2) and unicarpellate (1) gynoecia. Scale bar = 150 μm. **C**. Early stages of development of flowers in the intermediate zone. 1, unicarpellate female flower. 2, bicarpellate female flower. 3, atypical flower with incompletely formed gynoecium (center). 4, atypical flower with 4 staminodes. 5–7, Sterile male flowers (synandrodes) with 4 and 5 staminodes respectively. Scale bar = 150 μm. **D**. Intermediate zone showing atypical flowers. 1–2, sterile male flowers with 5 and 4 staminodes respectively. 3, Sterile male flower with incompletely fused staminodes. 4, Typical synandrode. Scale bar = 300 μm. **E**. Atypical flowers with rudimentary gynoecia (arrowheads) surrounded by 4 to 5 staminodes. Scale bar = 150 μm. **F**. Atypical sterile flowers with incompletely developed staminodes surrounding an undetermined structure (asterisk). Scale bar = 300 μm.

The initiation of atypical flowers in the intermediate zone is similar to that of female and male flowers (Figure 6A). In the intermediate zone, on nearly all inflorescences we observe a morphological gradient from typical female flowers to male flowers: female unicarpellate with free staminodes → sterile female with free staminodes → sterile male with free staminodes → sterile male with fused staminodes → incomplete synandria. Atypical flowers in the intermediate zone do not develop fully. They remain more or less rudimentary.

For example, Figure 6A shows atypical female flowers (1 and 2) with a rudimentary gynoecium and three staminodes. In Figure 6B, at the border between the female zone and the intermediate zone, the female flowers shift from a bicarpellate gynoecium (2) to a monocarpellate one (1). Towards the male zone, atypical flowers composed of four or five staminodes surrounding a rudimentary gynoecium are visible (Figure 6C, flower 3; 6E) and flowers consisting of three to four staminodes fused at the base, look like aborted synandrodes (Figures 6C, flower 4). Near the male zone, underdeveloped synandrodes are visible Figure 6C; flowers 5–7). However, on some inflorescences, there are atypical flowers consisting of four or five free staminodes (Figure 6D, flowers 1, 2) followed by synandrodes with incompletely fused staminodes (Figure 6D, flower 3), and atypical flowers that look like an intermediate between a synandrode and a synandrium (Figure 6D, flower 4). The continuous transition between female and male flowers in the intermediate zone is confirmed by the presence of very aberrant structures characterized by heterogeneous features. This is well represented in Figure 6F, where we observe two flowers consisting of a whorl of four aborted staminodes surrounding a structure (*) that can be viewed as an aborted gynoecium or another staminode.

## Discussion

Subfamily Aroideae is characterized by the presence of unisexual flowers, with the exception of the genus *Calla* (Cusimano et al. 2011). However, in several genera within the subfamily, flowers with male and female organs are often found in the intermediate zone of the spadix located between the male and female zones (Barabé, 2013). In *Dieffenbachia* the zone of atypical flowers is more extensive than in *Philodendron* and *Cercestis*, and there is a continuous morphological progression from female flowers to synandria. The atypical flowers located in the intermediate zone range in morphology from aborted female flowers to rudimentary sterile male flowers with incomplete staminodes. Starting from the female zone, the following sequence can be observed: typical female flowers with two carpels → female flowers with one carpel → sterile male flowers with free staminodes → synandrode → incomplete synandria → synandria. From a developmental point of view, the atypical flowers consist of a variable number of female, male and sterile appendices. Although atypical flowers are distant on mature spadices, during early stages of development, all types of atypical structures are closely packed. During their initial stages of development, flowers located in the intermediate zone experience two different morphogenetic constraints: one due to the female zone located in the lower part of the spadix and the other one due to the male zone located above the intermediate zone Barabé et al. (2004a, b, c, 2008). It is the longitudinal elongation of the inflorescence that brings about the isolation of atypical flowers from the other zones of the spadix.

The development of synandria was recently studied in the genus *Syngonium* (tribe Caladieae) (Barabé et al., 2012). In *Syngonium*, during early stages of development of the inflorescence, a few atypical synandrodes between the female zone and the male sterile zone are recognizable by their asymmetrical disorganized appearance. However, there are no atypical bisexual flowers as seen in *Cercestis* and *Dieffenbachia*. In *Syngonium*, all the atypical flowers located at the base of the sterile zone correspond to sterile male flowers and result from a more or less random disorganization of the typical structure of a synandrode. This disorganization is believed to be related to the proximity of these flowers to the female zone, adjacent to the lower row of synandrodes (Barabé et al., 2012).

Even though the stamens can be free in the genus *Gorgonidium*, the presence of a synandrium is a common character in Spathicarpeae (Table 1). Synandria have also been documented in tribe Caladieae (Barabé et al., 2012; Cusimano et al. 2011; Gonçalves et al., 2007). How similar is the development of synandria in both tribes?

In synandria of *Syngonium*, the fusion of stamens is less pronounced than in *Dieffenbachia*. In *Syngonium* the stamens are united internally and laterally to their neighbours and a cavity at the center of the synandria remains visible on mature flowers, where the connective is clearly visible. Individual stamens can also be observed in some synandria (Barabé et al., 2012). During early stages of development, the stamen primordia are more individualized in *Syngonium* (Figures Fifteen –Eighteen in Barabé et al., 2012) than in *Dieffenbachia* (Figure 6B, C). However in the genera *Gorgonodium* and *Croatiella* the stamens may be free or connate to different degrees in a synandrium (Table 1; Bogner and Nicolson, 1988; Gonçalves, 2005). This indicates that there are certainly different modes of development of synandria within the *Spathicarpeae* corresponding to various degrees of fusion of stamens. One can hypothesize that there is a unique process of formation of synandria varying in intensity between flowers with partially united stamens to fully developed synandria.

Gonçalves et al. (2007) suggested that the presence of staminodes associated with female flowers is a synapomorphy within Spathicarpeae. For these authors it is not possible to determine whether in *Bognera* Mayo and Nicolson the absence of staminodes is a plesiomorphy or an autapomorphy (secondary loss). The comparative floral development of *Bognera* with other genera having staminodes in the floral zone (e.g. *Homalomena*, *Schismatoglottis*, and other *Spathicarpeae)* will certainly help to resolve this question.

In certain species of *Schismatoglottis*, inter-pistillar staminodes are found at irregular intervals between female flowers (Barabé et al. 2004b). However, the absence of data related to the very early stages of development of the female flowers and staminodes in this genus prevents us from making detailed comparisons of floral development between *Schismatoglottis* and *Dieffenbachia*. In *Dieffenbachia* there is no discoid basal portion on female floral primordia. This is different from what was reported in female flowers of *Culcasia* (Barabé and Bertrand, 1996) and *Schismatoglottis* (Barabé et al., 2004b). On the other hand, the development of the female flower of *Dieffenbachia* presents some similarities with atypical bisexual flowers of *Cercestis*. This is not surprising considering that in the tribe *Spathicarpeae* only the genus *Bognera* does not have staminodes surrounding the ovary. In the short intermediate zone of the spadix of *Cercestis*, one can find atypical bisexual flowers consisting of a rudimentary gynoecium surrounded by a whorl of five rudimentary staminodes (Barabé, 2013; Barabé and Bertrand, 1996). The organisation and morphology of these atypical bisexual flowers are similar to those depicted during early stages of development of the female flowers of *Dieffenbachia*. Bisexual flowers constitute a plesiomorphic character in the Aroid family and female flowers associated with staminodes appear in many clades. A few species of the genus *Zantesdeschia* which makes up the sister group to *Spathicarpeae* (Cusimano et al., 2011) have a gynoecium surrounded by a whorl of three staminodes (Mayo et al., 1997). Also, in the genus *Synadrospadix,* Mayo et al. (1997) reported the presence of functional bisexual flowers in the intermediate zone of the spadix (Table 1). Therefore, from a developmental point of view, we believe that the presence of staminodes in the female zone constitutes a plesiomorphy instead of a synapomorphy as suggested by Gonçalves et al. (2007). Whether this is the case or not would need to be investigated at the molecular level.

**Figure 7:**
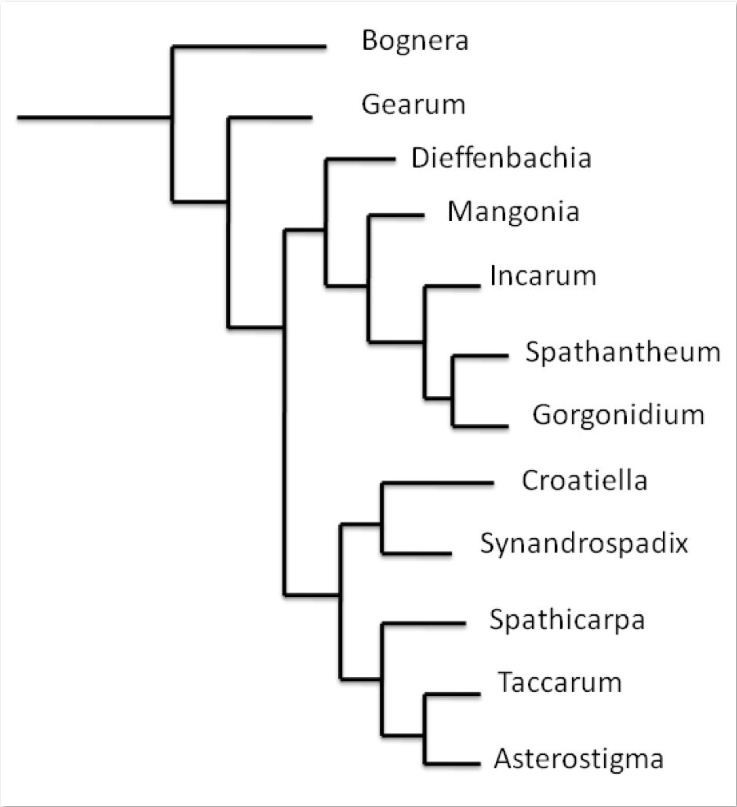
**Diagrammatic representation of phylogeny of the tribe** ***Caladieae*** **according to Cusimano et al. (** 2011 **) in their overall molecular phylogeny of the Araceae.**

*Dieffenbachia* forms a single clade with *Mangonia, Incarum, Spathantheum*, and *Gorgonidium* (Figure 7). However, neither of the inflorescence and floral characters (synandrium and synandrode) characterize this monophyletic clade (Table 1). It is not clear beyond that point if free stamens have evolved once or twice in the tribe *Spathicarpeae*, but both scenarios are possible.

Does the presence of synandria and synandrodes in Aroideae constitute a symplesiomorphic or apomorphic character? In the Aroideae, male flowers that form synandria have arisen several times in different tribes or clades. We believe that floral development is an important first step to help us to interpret the evolution of this character in the subfamily Aroideae. During the development of synandria and synandrodes in this group, more or less free primordia can be indentified during early stages of development. During subsequent stages of development, these primordia form a ring leading to the development of a synandrium or a synandrode. Given that during early stages of development one may recognize free primordia, this may indicate that in the Aroideae free stamens or staminodes represent a plesiomorphic condition.

## Conclusion

In conclusion, the structure of the atypical sterile male flowers of *Dieffenbachia* shows that the floral primordia in the intermediate zone experience developmental constraints during their early stages of development most likely as a result of the proximity of female flowers at the basal part of the spadix and male flowers in the upper portion. This would need to be confirmed using molecular approaches. The mode of development of different types of atypical flowers in Aroideae is linked to the morphogenesis of typical unisexual flowers, which represents a phylogenetic constraint channelling the nature or potential of atypical organs that can develop (Barabé 2013).

## Authors’ original submitted files for images

Below are the links to the authors’ original submitted files for images. Authors’ original file for figure 1 Authors’ original file for figure 2 Authors’ original file for figure 3 Authors’ original file for figure 4 Authors’ original file for figure 5 Authors’ original file for figure 6 Authors’ original file for figure 7
