# Supplementary figures and images for: Floral development of Dieffenbachia and the occurrence of atypical flowers in Araceae
Source: Bot Stud. 2014 Mar 14;55:30. doi: 10.1186/1999-3110-55-30 (PMC5432749; doi:10.1186/1999-3110-55-30)

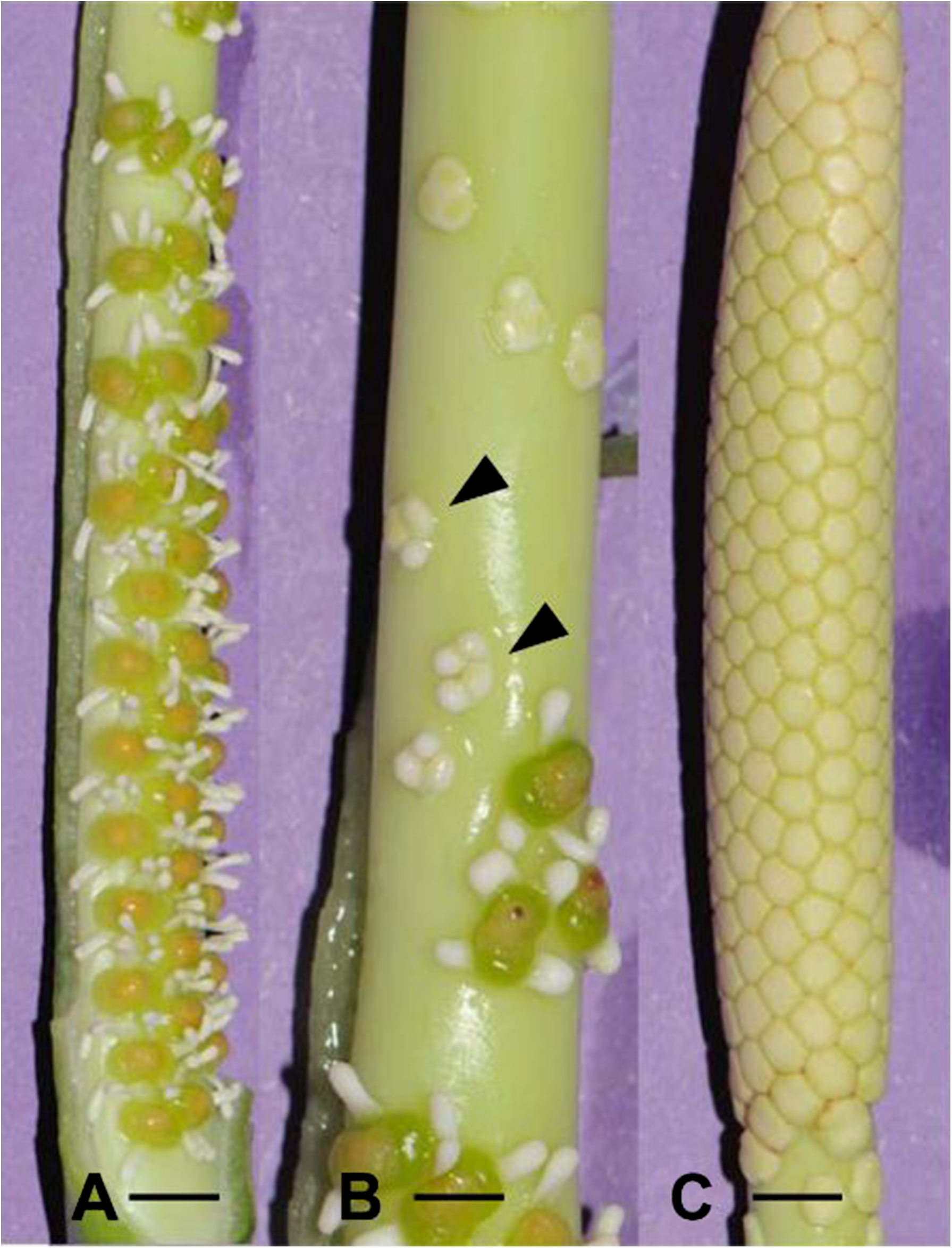

Supplement: Supplementary file 1 — Authors’ original file for figure 1 [file 40529_2014_86_MOESM1_ESM.tif]

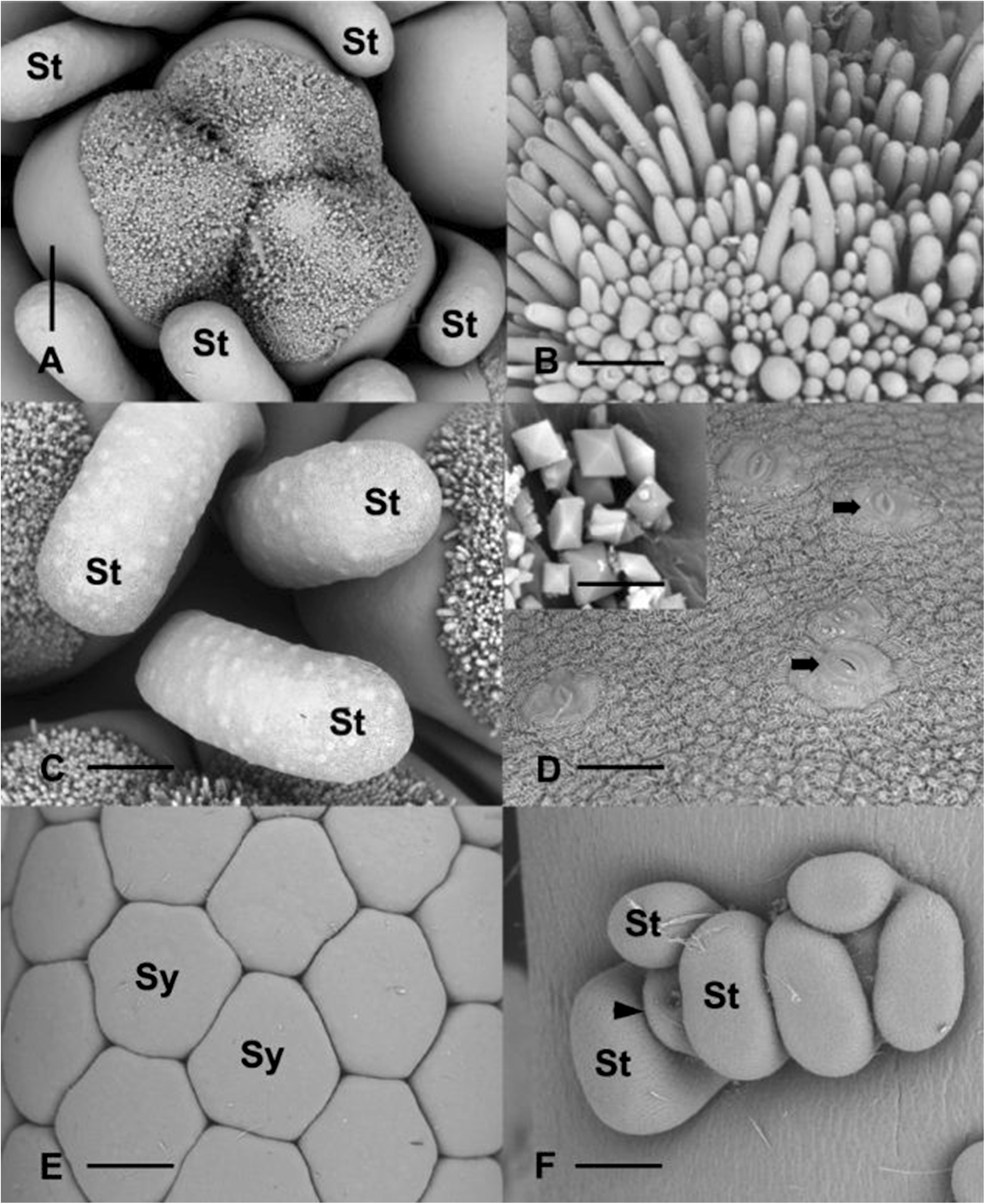

Supplement: Supplementary file 2 — Authors’ original file for figure 2 [file 40529_2014_86_MOESM2_ESM.tif]

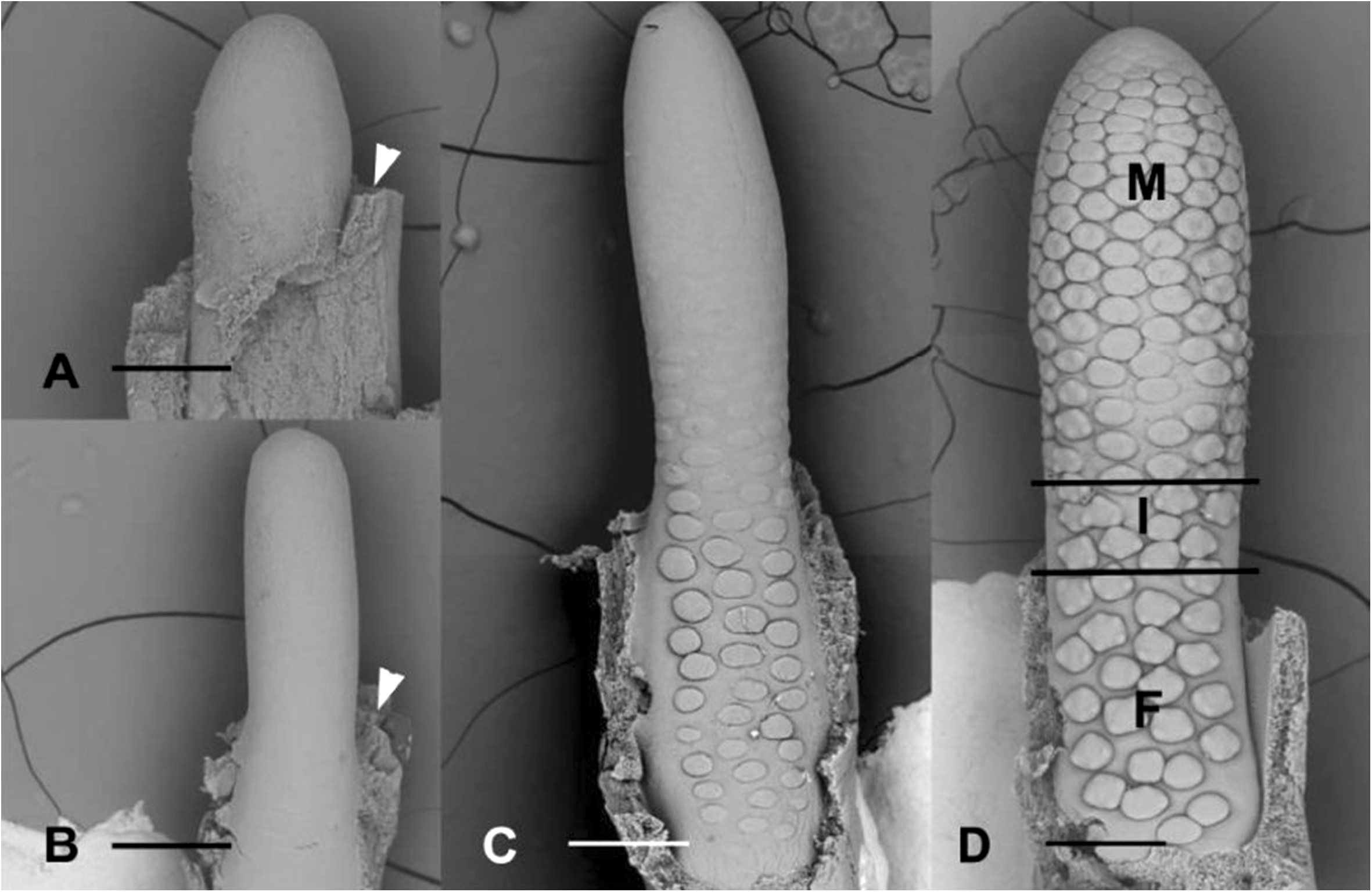

Supplement: Supplementary file 3 — Authors’ original file for figure 3 [file 40529_2014_86_MOESM3_ESM.tif]

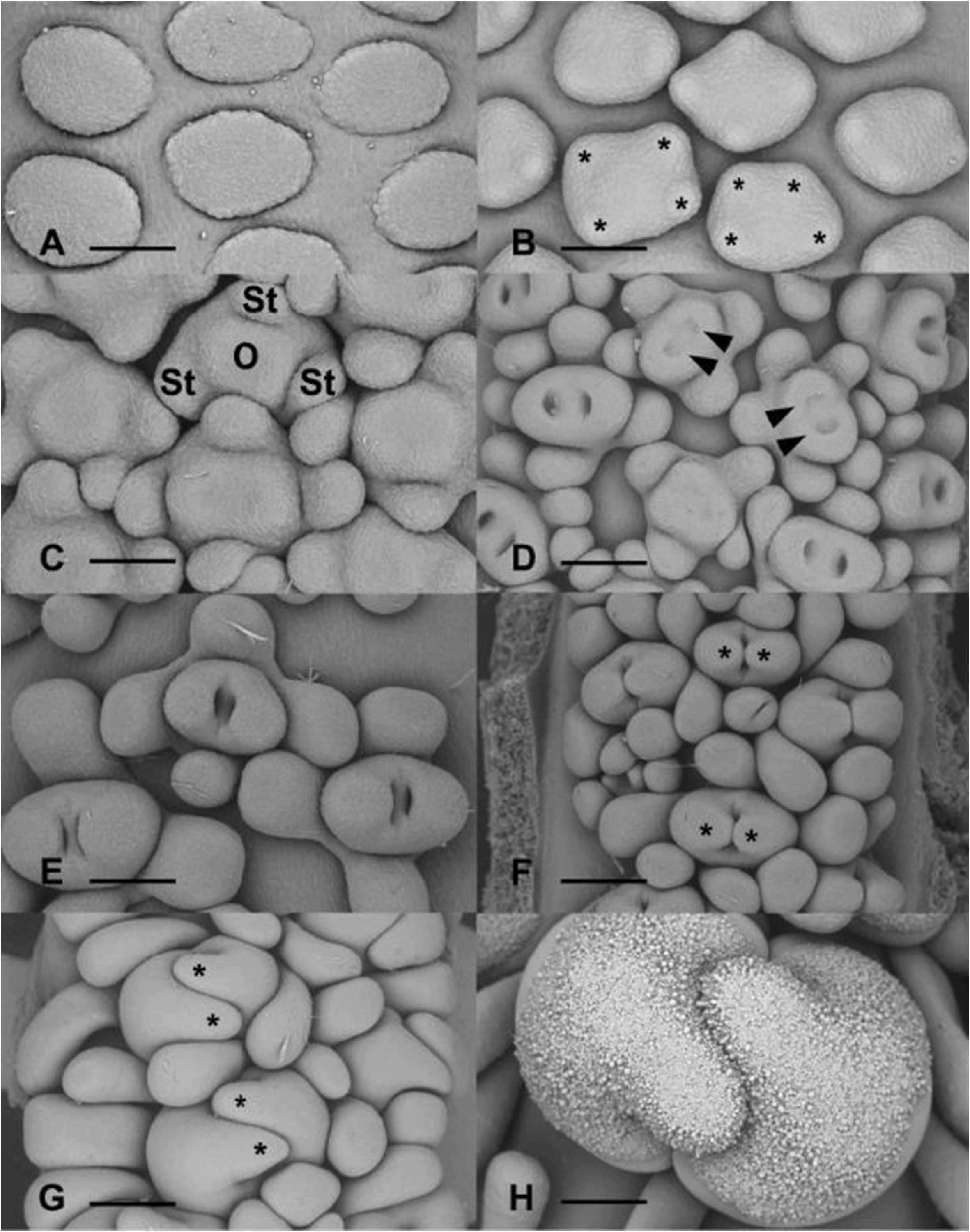

Supplement: Supplementary file 4 — Authors’ original file for figure 4 [file 40529_2014_86_MOESM4_ESM.tif]

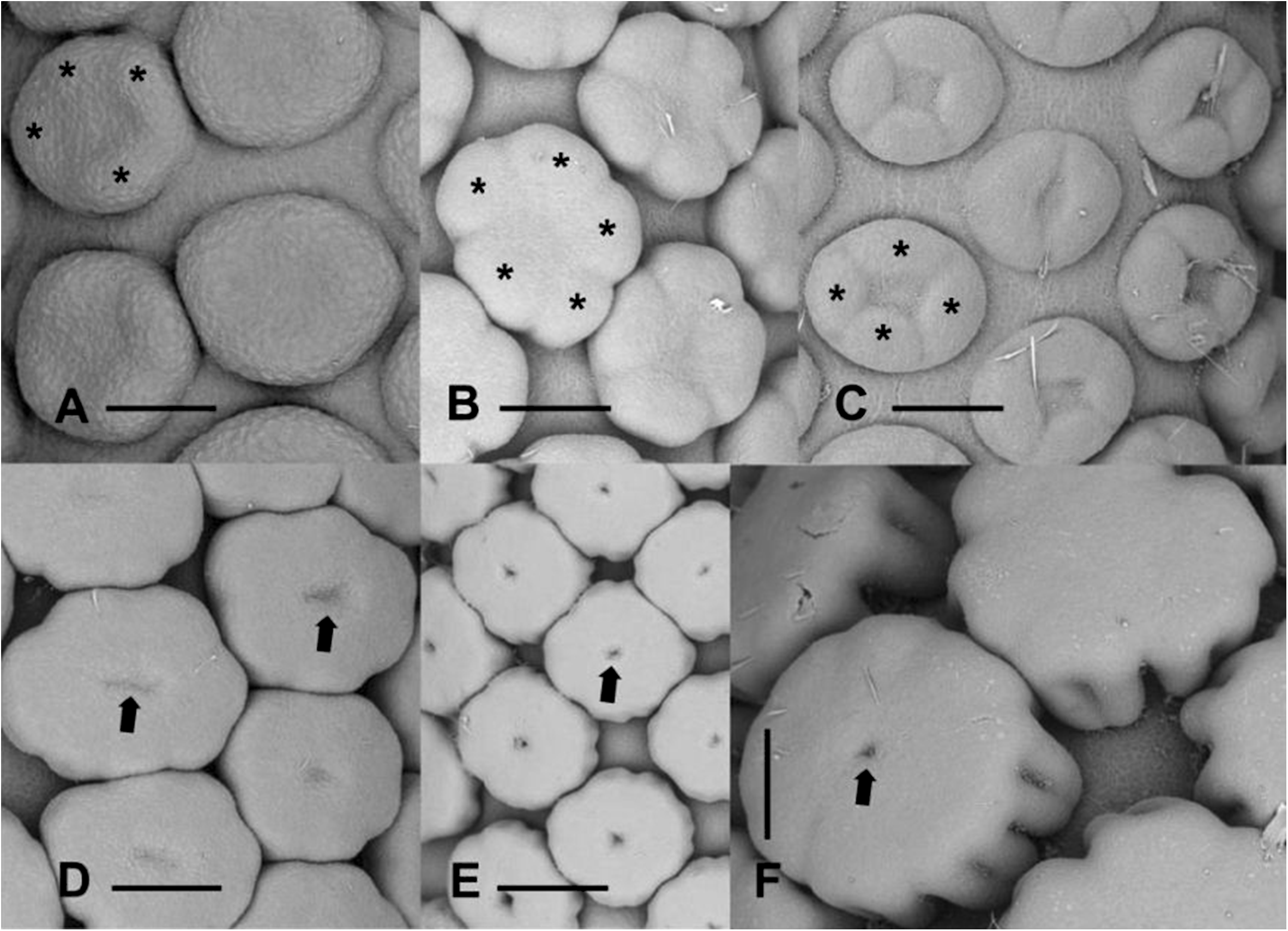

Supplement: Supplementary file 5 — Authors’ original file for figure 5 [file 40529_2014_86_MOESM5_ESM.tiff]

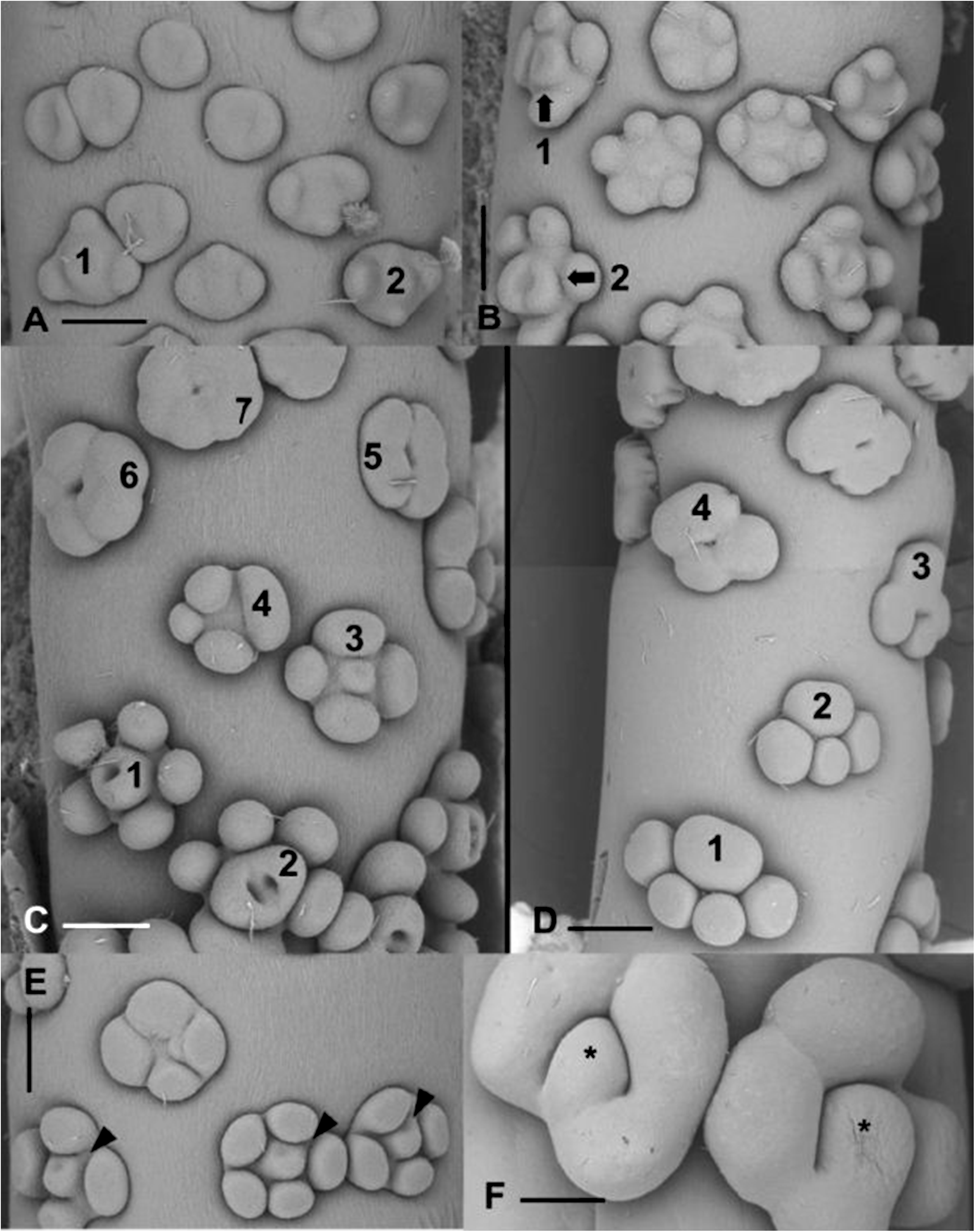

Supplement: Supplementary file 6 — Authors’ original file for figure 6 [file 40529_2014_86_MOESM6_ESM.tiff]

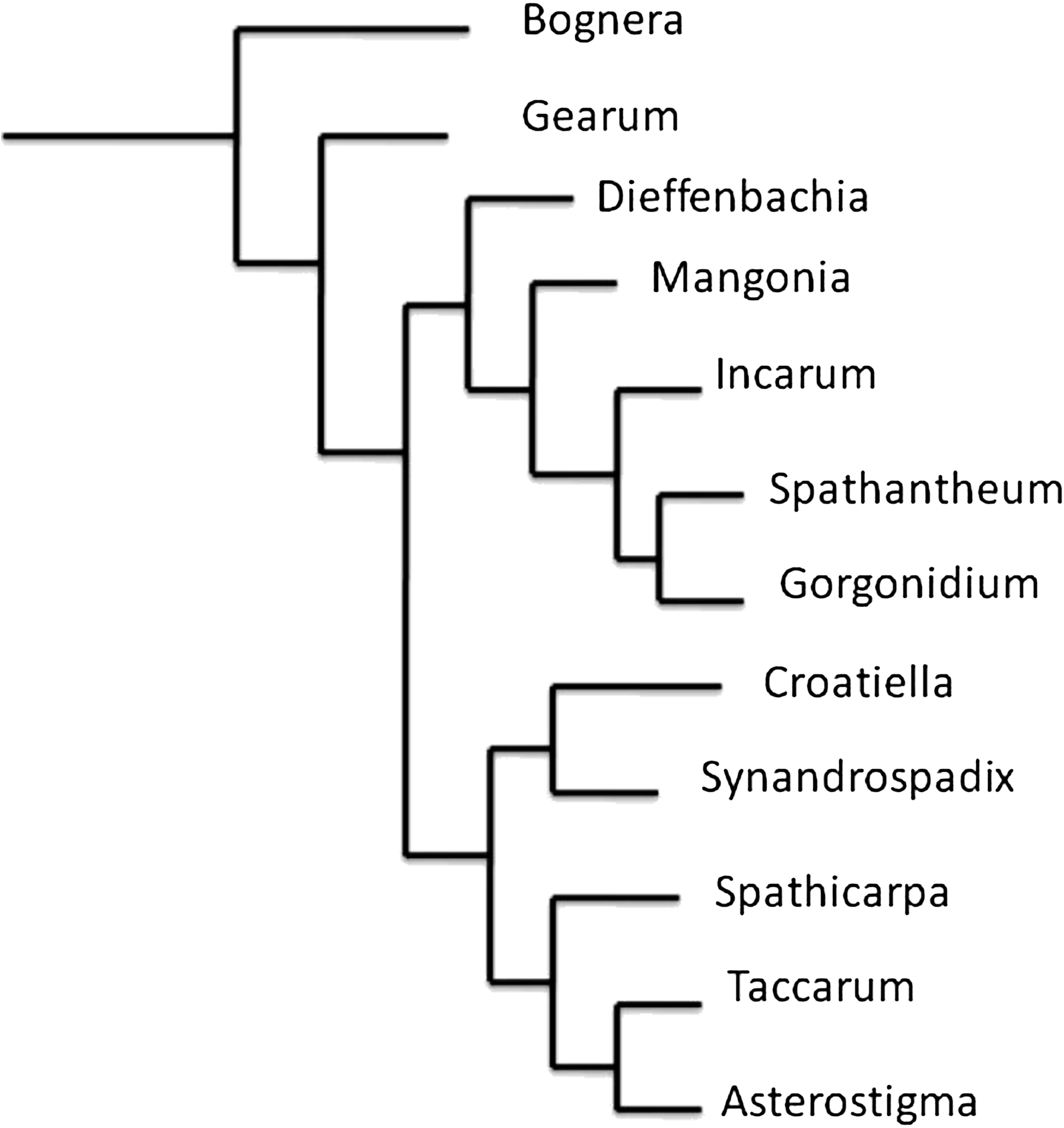

Supplement: Supplementary file 7 — Authors’ original file for figure 7 [file 40529_2014_86_MOESM7_ESM.tif]
